# Trichobezoar-Induced Small Bowel Obstruction in a Pediatric Patient: A Report of a Rare Case

**DOI:** 10.7759/cureus.85030

**Published:** 2025-05-29

**Authors:** Kumarie Budhu, Shamsa M Qaadri, Elizabeth Mathew, Louisdon Pierre, Brian Gilchrist, Noah Kondamudi

**Affiliations:** 1 Department of Pediatrics, The Brooklyn Hospital Center, New York, USA; 2 Department of Surgery, The Brooklyn Hospital Center, New York, USA

**Keywords:** abdominal ct scan, abdominal mass, exploratory laparotomy, small bowel obstruction, trichobezoar

## Abstract

We report a rare case of a four-year-old boy with small bowel obstruction caused by a trichobezoar. The initial diagnosis in the emergency department was acute gastroenteritis. The lack of improvement and the onset of bilious vomiting associated with abdominal distention raised the suspicion of bowel obstruction. Abdominal X-ray showed dilated loops of bowel, and a CT scan of the abdomen revealed a partially peripherally calcified mass containing debris as a cause of proximal small bowel obstruction. At exploratory laparotomy, a trichobezoar was visualized and removed, relieving the obstruction. The contents confirmed to be agglutination of ingested materials that are insoluble and consistent with hair. Trichobezoar, also known as a “hairball,” is described predominantly among females, often with a psychiatric history. We report a rare case of small bowel obstruction due to trichobezoar in a male toddler with no behavioral or psychiatric illnesses.

## Introduction

Pediatric small bowel obstruction (SBO) is a surgical emergency, presenting with bile-stained vomiting, abdominal pain, and distention [1]. Delays in recognition and intervention can result in significant morbidity and mortality, ranging from intestinal ischemia and necrosis [2]. In the US, common causes of SBO include congenital anomalies of the gastrointestinal tract (malrotation, Hirschsprung's disease, intestinal atresias), intussusception, incarcerated hernias, complications of acute appendicitis, and postoperative adhesions [3]. Less common acquired causes include magnet ingestions, ingestion of foreign bodies (magnet, water beads), and trichobezoars. Trichobezoars are ingested hairballs commonly located in the gastrointestinal tract that may be asymptomatic or manifest with abdominal pain and vomiting [4]. It is usually associated with trichotillomania (compulsive hair pulling) and trichophagia (compulsive hair ingestion) [5]. Rapunzel syndrome is a rare condition where the trichobezoar extends with a tail into the small intestine, also described in predominantly females [6]. Rare occurrences in men are related to past medical history of post-traumatic stress disorders and personality disorders with cluster A and B traits [7]. Trichobezoars are rare in children, particularly among preschoolers and especially among boys [8]. Case reports of trichobezoars among children predominantly involve females [9]. A 120-year systematic review of trichobezoar cases identified 19 (5%) males compared to 318 among female children [7]. Most of the previous reports of trichobezoar in male children were associated with behavior issues, mental retardation, or gastrointestinal tract abnormalities. We report an unusual case of trichobezoar causing SBO in an otherwise healthy boy.

## Case presentation

A four-year-old previously healthy boy presented to the emergency department (ED) with fever (101°F), non-bilious and non-bloody vomiting, and abdominal pain for one day. His last bowel movement, which was of normal consistency, was three days prior. He had reduced urinary output and felt increasingly tired. The physical exam was significant for sunken eyes and dry mucous membranes. The rest of the examination was unremarkable except for mild diffuse tenderness. The ED assessment was acute gastroenteritis with dehydration. Lab evaluation with complete blood count and serum electrolytes was within the normal range (Table 1).

**Table 1:** Lab data PT: Prothrombin time; PTT: Partial thromboplastin time; INR: International normalized ratio; BUN: Blood urea nitrogen; AST: Aspartate transferase; ALT: Alanine transaminase

Lab Test	Patient Results	Reference Range
Hemoglobin	12.4 g/dL	13.1-15.5 g/dL
Hematocrit	38%	39-47%
WBC	10.0 k/cmm	4.0-12 k/cmm
Platelet Count	297 k/cmm	130-400 k/cmm
PT	11.3 sec	9.5-12.1 sec
PTT	25.8 sec	23.9-30.7 sec
INR	1.0	0.9-1.1
Sodium	140 mmol/L	135-145 mmol/L
Potassium	3.7 mmol/L	3.5-5.1 mmol/L
Chloride	102 mmol/L	98-107 mmol/L
Bicarbonate	20 mmol/L	22-29 mmol/L
BUN	17 mg/dL	7-26 mg/dL
Creatinine	0.5 mg/dL	0.7-1.3 mg/dL
Random Serum Glucose	103 mmol/L	275-293 mmol/L
Calcium	10.3 mg/dL	8.4-10.2 mg/dL
Bilirubin Total	0.5 mg/dL	0.2-1.2 mg/dL
Bilirubin Direct	0.2 mg/dL	0.0-0.5 mg/dL
AST	23 U/L	8-34 U/L
ALT	10 U/L	6-55 U/L
Albumin	5.4 g/dL	3.5-5.0 g/dL
Alkaline Phosphatase	194 U/L	<500 U/L
Total Serum Protein	7.8 g/dL	6.4-8.3 g/dL

**Figure 1:**
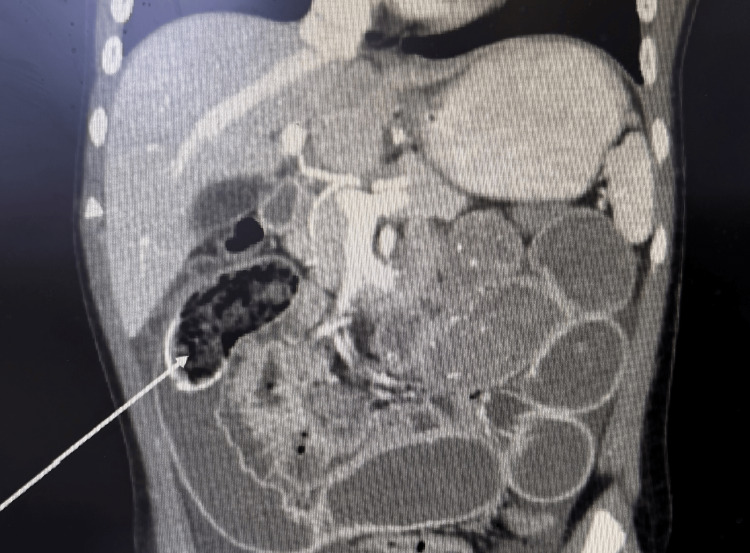
CT abdomen showing the trichobezoar (white arrow)

Attempts to hydrate orally were unsuccessful despite ondansetron administration, necessitating intravenous fluids. A diagnostic ultrasound performed at this time did not reveal any abnormalities. While being hydrated in the ED, the patient developed two episodes of non-projectile bilious vomiting, and the abdomen appeared to be distended with no change in bowel sounds. Abdominal X-ray at this time revealed dilated loops of bowel suspicious for small bowel obstruction. A CT scan of the abdomen and pelvis (Figure 1) performed due to persistent vomiting revealed a proximal SBO with a transition point in the right upper quadrant. An intraluminal mass (3.2 cm) consisting of peripherally calcified debris and air was identified in the right upper quadrant.

**Figure 2:**
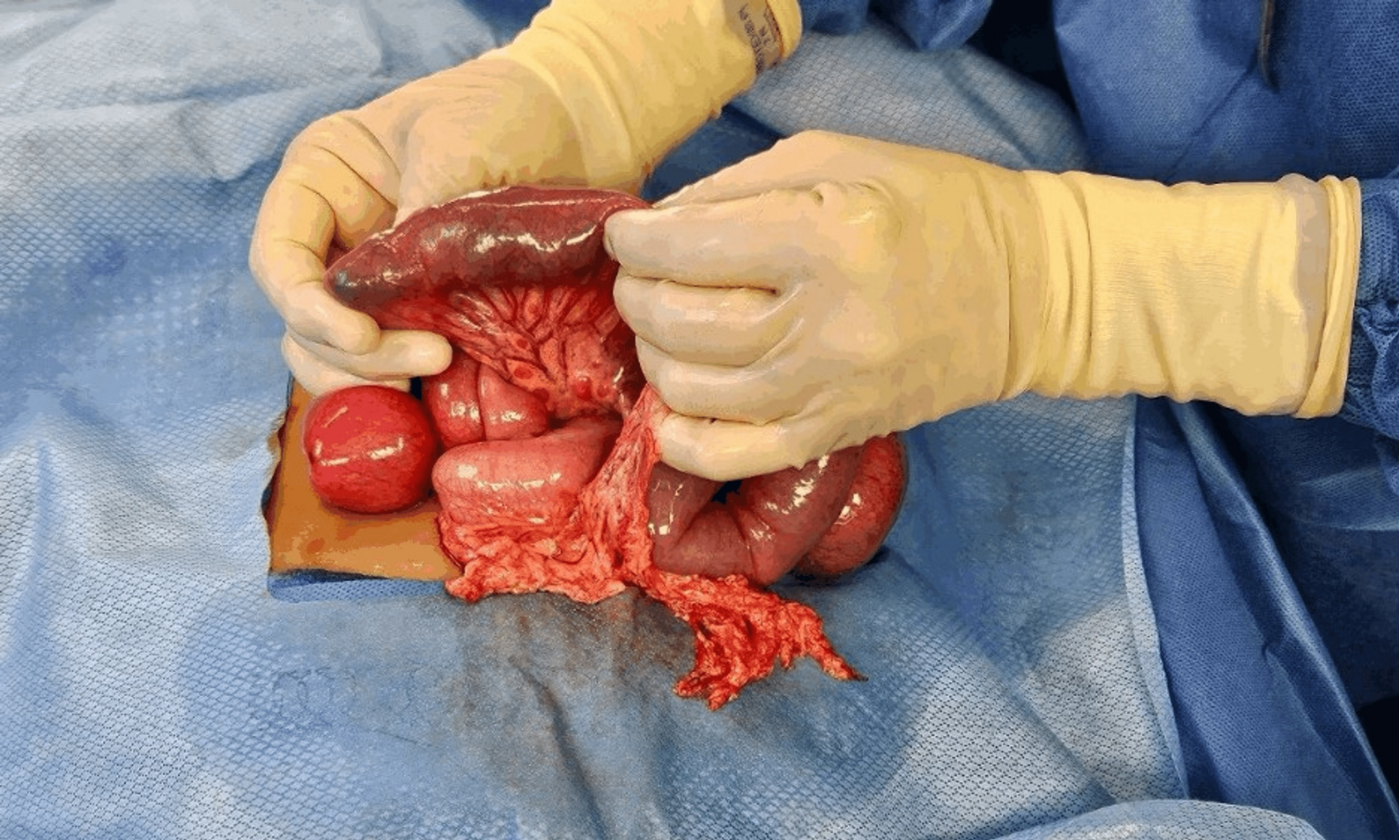
Mass in the intestinal segment

**Figure 3:**
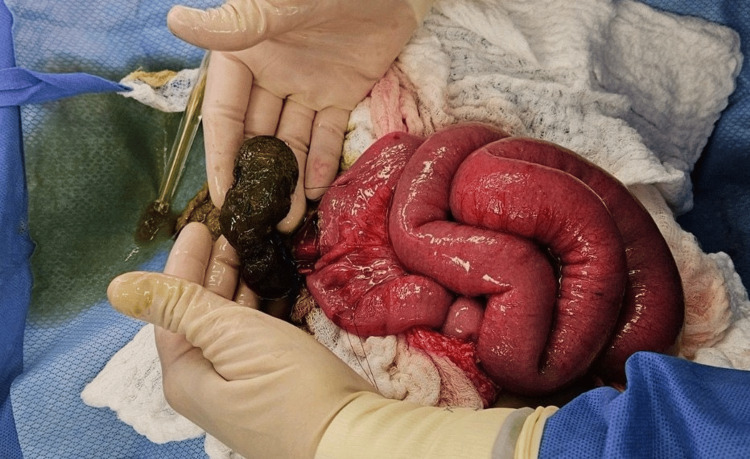
Image showing the trichobezoar removed from the gut

During surgery, a mass was noted within the intestinal lumen (Figure 2). The patient had the bezoar surgically removed (Figure 3), resulting in full recovery. The patient was discharged home on day 5 without any sequelae.

The official pathology report confirmed the diagnosis of trichobezoar. After recovery, the child admitted to eating his hair for no particular reason. He had no clinical features suggestive of any behavior or mental illness. The patient’s parents preferred long hair for their child and had refused all previous haircuts. They were unaware of the child’s propensity to eat his hair. They have since cut their hair and have kept it short. At the one-month follow-up, parents reported no new symptoms, and the patient had stopped eating his hair.

## Discussion

SBO among children is a surgical emergency of varied etiology as illustrated in Table 2.

**Table 2:** Causes of bowel obstruction in children Credit: The table was created by the authors.

Age Group	Condition
Neonatal (0-1 month)	Duodenal atresia
	Jejunal and ileal atresia
	Malrotation with midgut volvulus
	Necrotizing enterocolitis
Infants (1-12 months)	Intussusception
	Malrotation with midgut volvulus
Children (>1 year)	Incarcerated inguinal hernia
	Adhesions
	Acute appendicitis
	Meckels diverticulum
	Foreign body ingestions (bezoars)
	Inflammatory bowel disease

Among the uncommon causes of SBO is trichobezoar. A bezoar is an agglutination of undigestible ingested material in the digestive tract, most commonly in the stomach. These gastric bezoars are rare and can be distinguished based on the composition of the non-digestible ingested materials into four major types, such as phytobezoars, trichobezoars, pharmacobezoars, and lactobezoars (Table 3) [10].

**Table 3:** Gastric bezoars classification Credit: The table was created by the authors.

Bezoar Type	Composition	Classic Presentation
Phytobezoar	Indigestible food (vegetable matter, plant, or fruit fibers). Subtype: diospyrobezoar (persimmon fruit)	The most common type. Usually in adults. Nausea, vomiting, and gastric outlet obstruction.
Trichobezoars “hairball”	Hair (most common), fur, or wool	Usually in young females with psychiatric disorders (trichotillomania and trichophagia). Insidious onset. Nausea, epigastric pain, and gastric outlet obstruction.
Pharmacobezoars	Ingested medications (i.e., sucralfate, extended-release drugs, enteric-coated aspirin)	Signs and symptoms of gastric outlet obstruction.
Lactobezoar	Undigested milk concretions and mucus components	Usually, in premature infants or newborns with abdominal distention, irritability, vomiting, and feeding intolerance.

Bezoars may be asymptomatic or may present with nonspecific abdominal pain, abdominal distention, nausea, non-bilious vomiting, and constipation. Progression or migration of the bezoar into the small bowel may result in SBO. Delayed recognition and treatment of SBO can result in bowel ischemia and perforation [11]. Most bezoars are often small and singular, but multiple larger bezoars have been reported [12]. In Rapunzel syndrome, the trichobezoar crosses the pylorus and travels to the duodenum, ileum, and colon. Chin and Ng [13] described a rare case where a trichobezoar had descended into the cecum and the ascending colon. Bezoars can additionally cause intussusception [14] and pancreatitis [15].

To the best of our knowledge, we found no previous reports of trichobezoar causing SBO in a preschool boy. Our patient initially presented with a clinical picture of gastroenteritis. The lack of improvement over several hours and progression to bilious vomiting with abdominal distention resulted in further workup and detection of SBO. Exploratory laparotomy detected the site of obstruction in the small bowel and the presence of the trichobezoar in the intestinal lumen. It was not until recovery that there was an acknowledgment that the patient was pulling his hair (trichotillomania) and eating it (trichophagia). Our patient had no features of any behavioral or mental illness. The diagnosis of trichotillomania is challenging to make in preschool children [16]. Ultrasonography can detect bezoars [17] but is not always reliable. Diagnosis is often made by CT abdomen, gastrointestinal endoscopy, or laparotomy (as in our case). Management options of the bezoar (conservative versus surgical) vary depending on the clinical presentation, number, size, and composition. Conservative management includes IV hydration and prokinetic agents to facilitate the passage of the bezoar through the GI tract [18]. Ladas et al. [19] in their systematic review of over two dozen research articles involving 46 patients concluded that Coca-Cola alone was effective in the dissolution of at least one-half of cases of gastric phytobezoars, and combined with endoscopic fragmentation techniques, the success rates were nearly 90% without the need of surgical intervention. Wang et al. evaluated the indications for surgery in the treatment of bezoar-induced SBO by surveying 40 adult patients. They concluded that a large bezoar size and high CT values are indications of surgery, especially after ineffective nonsurgical treatments [20]. Timely recognition and surgical intervention can prevent complications and reduce mortality.

As trichobezoars are associated with behavioral and mental illnesses, it is imperative to obtain a comprehensive past medical and social history with a special focus on behavioral and psychiatric symptoms. Recurrence [20] of trichobezoar following surgical removal can be up to 20%, underscoring the need for regular follow-up and appropriate behavioral interventions among affected children.

## Conclusions

We present a rare case of SBO due to trichobezoar in a preschool boy with no known behavioral or mental illnesses. Timely recognition and prompt surgery resulted in a good outcome for our patient. In hindsight, the long hair in this boy could have been a soft clue to consider trichobezoar as a possible cause of his SBO. This case illustrates that in children with long hair, trichobezoar can be a plausible cause of SBO, regardless of gender. In addition to treating underlying behavioral issues, appropriate simple interventions, such as keeping hair short, can help prevent recurrence.
